# Molecular dynamics of inflammation resolution: therapeutic implications

**DOI:** 10.3389/fcell.2025.1600149

**Published:** 2025-05-08

**Authors:** Amro M. Soliman, Mohamed Soliman, Syed Sajid Hussain Shah, Habeeb Ali Baig, Nawal Salama Gouda, Bandar Theyab Alenezi, Awwad Alenezy, Ahmed M. S. Hegazy, Muhammad Jan, Elhassan Hussein Eltom

**Affiliations:** ^1^ Department of Biological Sciences, Faculty of Science, Concordia University of Edmonton, Edmonton, AB, Canada; ^2^ Department of Microbiology, Faculty of Medicine, Northern Border University, Arar, Saudi Arabia; ^3^ Department of Pathology, Faculty of Medicine, Northern Border University, Arar, Saudi Arabia; ^4^ Department of Pharmacology, Faculty of Medicine, Northern Border University, Arar, Saudi Arabia; ^5^ Department of Family and Community Medicine, Faculty of Medicine, Northern Border University, Arar, Saudi Arabia; ^6^ Department of Anatomy, Faculty of Medicine, Northern Border University, Arar, Saudi Arabia

**Keywords:** acute inflammation, resolution, specialized pro-resolving molecules, proinflammatory pathways, resolution pharmacology

## Abstract

Inflammation is a critical part of innate immune response that is essential for exclusion of harmful stimuli and restoration of tissue homeostasis. Nonetheless, failure to resolve inflammation results in chronic inflammatory conditions, including autoimmune diseases. Conventionally, resolution of inflammation was deemed a passive process; however, evidence indicates that it entails active, highly regulated molecular and cellular events involving efferocytosis-driven macrophage reprogramming, post-transcriptional regulatory mechanisms and the production of specialized pro-resolving mediators (SPMs). These processes collectively restore tissue homeostasis and prevent chronic inflammation. Emerging therapeutic approaches targeting these pathways demonstrate promising results in preclinical studies and clinical trials, enhancing resolution and improving overall disease outcome. This resulted in a paradigm shift from conventional anti-inflammatory strategies to resolution-focused treatment. Yet, challenges remain due to the complexity of resolution mechanisms and tissue-specific differences. This review summarizes current advances in inflammation resolution, emphasizing emerging concepts of resolution pharmacology. By employing endogenous mechanisms facilitating resolution, novel therapeutic applications can effectively manage several chronic inflammatory disorders.

## 1 Introduction

Inflammation is a physiological immune response triggered upon injury and/or infection to eliminate harmful stimuli, promote tissue repair and establish immune memory for future encounters (Kumar et al., 2004). The acute inflammatory response involves a complex and coordinated series of molecular and cellular events, including release of soluble mediators such as chemokines, cytokines, free radicals and eicosanoids, regulating its initiation and resolution (Rankin, 2004). Traditionally, the termination of inflammatory responses was mainly credited to passive dissipation of pro-inflammatory inducers; however, the expanding literature implies inflammation resolution to be highly regulated. Canon of Medicine, compiled in 1025 by the physician-philosopher Avicenna, was first to highlight the concept of active resolution of inflammation, (Sina, 2005). In the late 1970s, Kumar et al reemphasized resolution as a distinctive process mediated by cellular and soluble effectors with well-characterized events (Kumar et al., 2021). Following that, key insights into cellular dynamics of inflammation resolution emerged following studies revealing that macrophages phagocytosed apoptotic polymorphonuclear cells (PMNs), as a key event in establishing resolution (Savill et al., 1989). Macrophages were subsequently recognized for their role in coordinating inflammation resolution processes. This was accentuated with complete resolution as the ideal inflammatory outcome, and chronic inflammation attributed to resolution failure (Cotran et al., 1999).

Discoveries of arachidonic acid metabolism and cytokine signaling enhanced our understanding of acute inflammation as a complex and biochemically driven process. The 1982 Nobel Prize was awarded to Bergström, Samuelsson and Vane for emphasizing the significance of prostaglandin biosynthesis and aspirin’s mechanism of action (Vane, 1971; Samuelsson, 1983). The work on arachidonic acid derivatives highlighted the role of lipid autacoids in inflammation induction and resolution (Samuelsson, 1983). The discovery of lipoxins (LXs) by Serhan et al. revealed their anti-inflammatory and pro-resolving activities, such as suppressing neutrophil recruitment and enhancing clearance of tissue debris by macrophages (Serhan et al., 1984; Serhan, 2005), emphasizing a dual effect of LXs in limiting neutrophil activity while promoting monocyte trafficking for tissue repair. As a result, LXs are currently perceived as active mediators of tissue restoration rather than passive anti-inflammatory agents (Maddox and Serhan, 1996). Additional molecules were reported over the years to be actively involved in inflammation resolution and were termed specialized pro-resolving mediators (SPMs). These mediators cemented the concept of resolution as a dynamic biologically orchestrated process crucial for restoring tissue homeostasis (Panigrahy et al., 2021).

Over the past two decades, significant progress has been made in understanding the resolution of cardinal signs of inflammation: heat (*calor*), pain (*dolor*), redness (*rubor*), swelling (*tumor*) and loss of function (*functio laesa*) (Heidland et al., 2006; Tracy, 2006). Interplay between effective resolution, innate immunity and adaptive immunity was reported, where unresolved acute inflammation led to maladaptive immune responses (Newson et al., 2014). This supported the idea that chronic inflammatory diseases may not only be driven by persistent pro-inflammatory processes but also by impaired resolution, with therapies aimed at activating resolution potentially guiding inflammation down a pro-resolution pathway. Unlike traditional anti-inflammatory approaches, pro-resolution strategies offer broader potential; however, challenges remain, such as defining the diverse tissue- and stimulus-specific nature of resolution pathways (Fullerton and Gilroy, 2016). Additionally, while central mediators of resolution have been identified, it is uncertain whether a single pro-resolution therapy can address multiple diseases.

The literature is progressively distinguishing the terms “anti-inflammation” and “pro-resolution,” emphasizing the concept that resolution is an active process, facilitating restoration of tissue homeostasis and the transition from innate to adaptive immunity (Serhan et al., 2007; Buckley et al., 2014). Efforts to target pro-resolution pathways in chronic inflammatory diseases are ongoing, with new therapeutic models being explored. This evolving understanding encourages a shift in how chronic inflammation is treated, suggesting that pro-resolving agents may be as effective, or even synergistic with, or superior to, conventional anti-inflammatory drugs (Fullerton and Gilroy, 2016). Although many effective anti-inflammatory treatments are available, including NSAIDs, anti-cytokine therapies and steroids, addressing underlying disease mechanisms remains a significant challenge. Diseases driven by persistent inflammation could be treated by activating pro-resolution pathways that are either pathologically suppressed or by enhancing functional pro-resolution mechanisms. To target resolution specifically, the aim should be to alter the trajectory of established inflammation-driven disease in a clinically relevant manner by leveraging endogenous “off switches,” such as signaling cascades or cellular interactions, that lead to inflammation resolution (Fullerton and Gilroy, 2016). This review offers an update on the field of inflammation resolution, focusing on key molecular and cellular participants and mechanisms that influence their behavior, fate and clearance while emphasizing ongoing efforts to develop therapies targeting pro-resolution pathways.

**FIGURE 1:**
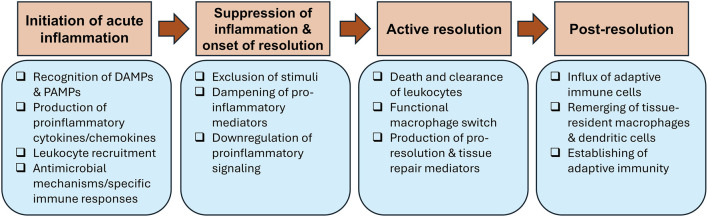
Steps of acute inflammation and its resolution. Acute inflammation is triggered after recognition of damage-associated molecular patterns (DAMPs) and pathogen-associated molecular patterns (PAMPs) by receptors expressed on resident immune cells. This results in the secretion of pro-inflammatory mediators that aid in recruitment of granulocytes to the inflammation site. Several antimicrobial mechanisms are deployed to remove the injurious stimuli, which kicks off the suppression of inflammation and onset of resolution by downregulating pro-inflammatory mediators and signaling. Active resolution is activated by efferocytosis, resulting in macrophage phenotypic switch from classically activated (M1) to alternatively activated (M2) macrophages and production of pro-resolving molecules. The post-resolution phase occurs after the completion of resolution, when adaptive immune cells infiltrate tissues to establish adaptive immune responses.

A typical acute inflammatory response and its resolution goes through four main phases that each comprises several critical events (summarized in Figure 1): 1) initiation of acute inflammation, 2) suppression of inflammation and onset of resolution, 3) active resolution and 4) post-resolution.

## 2 Initiation of acute inflammation

Following injury and/or infection, damage-associated molecular patterns (DAMPs) (Roh and Sohn, 2018), released by necrotic cells, and pathogen-associated molecular patterns (PAMPs) (Mogensen, 2009), including conserved motifs of invading pathogens, are detected by innate receptors such as toll-like receptors (TLRs) on tissue-resident cells. Recognizing DAMPs or PAMPs triggers the onset of an acute inflammatory response (Medzhitov and Janeway, 2000), leading to release of various pro-inflammatory mediators facilitating cellular recruitment and modulating immune responses to clear infections (Abdallah et al., 2017). Previously, we discussed mediators, cellular effectors and pathways involved during onset and peak phase of acute inflammation, and importantly demonstrated that mechanisms involved in induction of inflammation are evolutionarily conserved among cold- and warm-blooded animals (Soliman and Barreda, 2023). These mechanisms involve exudation of proteins, upregulation of cell adhesion molecules on endothelial cells and influx of granulocytes (PMNs in nonspecific inflammation or eosinophils in allergic responses) (Fullerton and Gilroy, 2016). Recruited immune cells act through intracellular mechanisms, such as superoxide radicals, myeloperoxidase, proteases and lactoferrins, or extracellularly via neutrophil extracellular traps (NETs) to neutralize pathogens (Schmid-Schönbein, 2006). Drugs such as NSAIDs and cytokine-neutralizing antibodies, e.g., tumor necrosis factor (TNF)-specific antibodies, are frequently utilized to suppress inflammation to treat chronic inflammatory diseases (Yeung et al., 2018).

## 3 Suppression of inflammation and onset of resolution

Inflammation resolution is triggered by effector cells that induce inhibition of pro-inflammatory profiles and transition toward tissue repair and homeostasis (Buckley et al., 2014). Indeed, the process depends on the extent to which inflammatory cells have neutralized provocative stimuli (Buckley et al., 2014). The transition from pro-inflammatory to anti-inflammatory profile and subsequent restoration of tissue homeostasis, traditionally referred to as resolution, is now understood to be an active process rather than a passive dissipation of inflammation (Buckley et al., 2013). Initially, dampening of acute inflammation starts only once the injurious agents responsible for triggering the inflammatory response are eliminated (exclusion of the stimuli). Afterward, the production of pro-inflammatory mediators ceases, and any remaining mediators are degraded and cleared (dampening of pro-inflammatory mediators). This results in the downregulation of pro-inflammatory signaling and halting further leukocyte recruitment and edema formation.

### 3.1 Exclusion of stimuli

For resolution to proceed effectively, agents that triggered the inflammatory response must be cleared. We previously highlighted antimicrobial responses by which the immune system can remove bacterial infections (Soliman et al., 2021; Soliman and Barreda, 2022; Soliman and Barreda, 2023). These include NADPH oxidase-dependent killing by PMNs, antimicrobial peptides, phagocytosis and NETs. Additionally, leukocytes, such as neutrophils, engulf debris, clearing the injury site of dead tissue and creating pathways for angiogenesis necessary for restoring tissue repair (Peiseler and Kubes, 2019). Dysregulated inflammatory responses in the case of immunodeficiency disorders (e.g., chronic granulomatous disease) have been attributed to defects in mechanisms of bacterial clearance (Pollock et al., 1995), causing resolution failure. Likewise, autoimmune diseases such as rheumatoid arthritis are driven by persistent endogenous antigens (Firestein, 2003).

### 3.2 Dampening of pro-inflammatory mediators

After exclusion of stimuli, levels of pro-inflammatory mediators (cytokines, chemokines, eicosanoids and cell adhesion molecules) must return to their pre-inflammatory baseline. The depletion of chemokines through mechanisms such as proteolytic cleavage and sequestration is essential for creating a resolving environment and halting neutrophil influx (Ortega-Gómez et al., 2013). Prostaglandins contributing to vasodilation and edema formation were found to undergo catabolism during resolution (Hansen et al., 1999). Leukotrienes are degraded by β-oxidation pathway, breaking down carboxyl ends of molecules into short-chain metabolites (Hansen et al., 1999; Freire and Van Dyke, 2013).

Proteolysis of chemokines has been shown to be a key mechanism for their depletion. Matrix metalloproteinases (MMPs), known for their role in breaking down extracellular matrix proteins during many physiological and pathological conditions, contribute to modulating activity of several bioactive molecules involved during inflammation (Tam et al., 2004; Dean and Overall, 2007). Levels of molecules including chemokines (McQuibban et al., 2000; Moelants et al., 2013), defensins (Wilson et al., 1999), mannose-binding lectin (Butler et al., 2002) and TNF-α (Gearing et al., 1994) are controlled by MMPs during inflammation resolution. At the cellular level, macrophages regulate acute inflammatory responses by selectively cleaving chemokines via MMP-12 (Dean et al., 2008). For example, macrophage-derived MMP-12 targets ELR motif in CXC chemokines, a domain essential for receptor binding, depicting these chemokines ineffective (Dean et al., 2008). These findings emphasize these endogenous mechanisms clearing pro-inflammatory mediators and suggest that their dysregulation can lead to chronic inflammation. Indeed, leveraging pro-inflammatory catabolic pathways therapeutically could help shift ongoing inflammation toward resolution.

Chemokines drive cell migration via conventional G protein-coupled chemokine receptors. A subset of chemokines is recognized by a unique class of atypical chemokine receptors (ACKRs), previously referred to as decoys or chemokine-binding proteins, including chemokine-binding protein D6 and CCR5 (Ariel et al., 2006; Nibbs and Graham, 2013; Vacchini et al., 2016). Unlike conventional receptors, ACKRs do not induce leukocyte migration because they cannot activate classical G protein-dependent signaling pathways (Vacchini et al., 2016). These receptors bind ligands without triggering classical signaling pathways, acting as scavengers for pro-inflammatory signals. Instead, ACKRs sequester chemokines from the environment, which is critical for shaping chemokine gradients (Nibbs and Graham, 2013). As a result, ACKRs are increasingly recognized as vital regulatory elements in chemokine networks across various physiological and pathological conditions. For example, CCR5 expressed on apoptotic neutrophils can scavenge chemokines CCL3 and CCL5 in mice (Ariel et al., 2006). In *Ccr5* ^−/−^ mice, CCL3 and CCL5 were elevated in peritoneal exudates during the resolution of acute peritonitis (Ariel et al., 2006).

### 3.3 Downregulation of pro-inflammatory signaling

After removal of stimuli and lowering levels of pro-inflammatory mediators at the inflammation site, various pro-inflammatory receptor families and their signaling pathways are deactivated to prevent collateral damage from a sustained pro-inflammatory state. A failure of this process would result in a cytokine storm that can occur even in the absence of positive bacterial blood cultures (i.e., sepsis), indicating excessive and prolonged activation of the innate immune system despite eliminating the initial trigger (Buckley et al., 2013). The “stop signals” of inflammation consist of negative feedback regulators. These signals counteract pro-inflammatory signaling pathways and suppress further production of pro-inflammatory mediators.

Inflammation is regulated by both transcriptional and post-transcriptional mechanisms, which control the expression of proteins involved in its initiation and resolution (Sugimoto et al., 2016). While transcription is the initial step in gene expression, post-transcriptional regulation is essential for rapidly suppressing inflammation by facilitating mRNA degradation. This is particularly important because mRNA can be long-lived, and simply halting its synthesis does not immediately stop inflammation. mRNAs of pro-inflammatory cytokines are frequently controlled through mRNA decay or blocking their translation (Anderson, 2008). These mechanisms protect the host from the pathological overexpression of inflammatory proteins. Many mRNAs contain adenine- or uridine-rich elements (AREs) within their 3′-untranslated regions (UTRs), which attract destabilizing factors and translational silencers (Anderson, 2008). Among the ARE-binding proteins that promote mRNA destabilization are tristetraprolin (TTP) (Carballo et al., 2000) and steroid receptor co-activator 3 (SRC3) (Li et al., 2012). Additionally, microRNAs (miRNAs) can post-transcriptionally regulate mRNA stability and translation, limiting the expression of inflammatory mediators. Although the precise role of post-transcriptional regulation in inflammation resolution is not entirely understood, it presents exciting opportunities for pharmacological interventions to address the overproduction of inflammatory proteins (Mazumder et al., 2010). Downregulators of pro-inflammatory signaling, summarized in Table 1, can be categorized into 1) anti-inflammatory mediators that can negatively regulate signaling of pro-inflammatory pathways and their activated transcription programs; 2) post-transcriptional regulation promoting mRNA degradation or inhibiting its translation; and 3) miRNAs.

**TABLE 1:** Inhibitors of pro-inflammatory signaling.

Category	Inhibitory agent	Targeted pro-inflammatory pathway/biological process	References
Anti-inflammatory mediators	LRRC33	TLR signaling and subsequent NF-κB activation	Liu et al. (2013)
	RP105 (CD180)		Divanovic et al. (2005)
	Deubiquitinase cylindromatosis	NF-κB	Kovalenko et al. (2003)
	PGE2	Expression of TLR4	Degraaf et al. (2014)
Post-transcriptional regulation by binding of destabilizing agents to mRNA resulting in its degradation	TTP	Expression of GM-CSF	Carballo et al. (2000)
		Expression of IL-2	Ogilvie et al. (2005)
		Expression of IL-6	Sauer et al. (2006)
		Expression of iNOS	Linker et al. (2005)
		Expression of COX2	Phillips et al. (2004)
		Expression of IFNγ	Ogilvie et al. (2009)
		Expression of TNF	Carballo et al. (1998)
Post-transcriptional regulation by binding of destabilizing agents to mRNA, inhibiting its translation	TIA1	Expression of TNF	Piecyk et al. (2000), Mukhopadhyay et al. (2009)
	SRC3 binds to TIA1, enhancing the attachment of TIA1 to ARE	Expression of TNF, IL-6, IL-1β	LI et al. (2012)
	GAIT complex	Expression of CCL22, CCR3, CCR4, CCR6	Piecyk et al. (2000), Vyas et al. (2009)
	ZC3H12A	NF-κB resulting in inhibition of macrophage activation, TNF and iNOS expression	Liang et al. (2008)
miRNA	miR-146	Translation of mRNA encoding IRAK1, IRAK2, TRAF6 required for NF-κB activation	Mukhopadhyay et al. (2009)
	miR-21	Translation of pro-inflammatory tumor suppressor PD4 that inhibits IL-10 expression	Sheedy et al. (2010)
	miR-9	TLR4-MyD88-NF-κB	Bazzoni et al. (2009)
	miR-155	TLR-signaling by targeting TAB2, MyD88, IKKε, RIPK1, C/EBPβ, eNOS and NF-κB subunit p65	Tili et al. (2007), Ceppi et al. (2009), Tang et al. (2010), Wu et al. (2014), Obora et al. (2017)
	miR-132	Expression of acetylcholinesterase	Shaked et al. (2009)
	miR-223	IKKα, attenuating TLR9/NF-κB signaling pathway in neutrophils Granzyme B, Roquin and STAT3	He et al. (2017)
	miR-17	TNF-α signaling by targeting TRAF2 and cIAP2	Akhtar et al. (2016)
	miR-18	Pias3 and TNFAIP3, negatively regulating Stat3 and NF-κB signaling	Brock et al. (2011)
	miR-23	IL-17-mediated TNF-α production, IL-1β-induced NF-κB signaling, through targeting IKKα, TAB2 and TAB3	Zhu et al. (2012)

AREs (adenylate- and uridylate-rich elements), COX2 (cyclooxygenase 2), CCL (CC-chemokine ligand), CCR (CC-chemokine receptor), C/EBPβ (CCAAT/enhancer-binding protein beta), GAIT (gamma-activated inhibitor of translation), GM-CSF (granulocyte-macrophage colony-stimulating factor). IFNγ (interferon gamma), IKKε (IκB kinase epsilon), and IKKα (IκB kinase alpha), IL (interleukin), iNOS (inducible nitric oxide synthase), IRAK (IL-1, receptor-associated kinases), LRRC33 (leucine-rich repeat-containing protein 33), MyD88 (myeloid differentiation primary response 88), NF-κB (nuclear factor kappa B), PD4 (programmed cell death 4), PGE2 (prostaglandin E2), RIPK1 (receptor-interacting protein kinase 1), RA (rheumatoid arthritis), CD180 (radioprotective 105), SRC3 (steroid receptor coactivator 3), STAT3 (signal transducer and activator of transcription 3), TAB (TAK1-binding proteins), TIA1 (T-cell intracellular antigen 1), TLR (Toll-like receptor), TNF (tumor necrosis factor), TNFAIP3 (TNF, alpha-induced protein 3), TRAF (TNF, receptor-associated factors), ZC3H12A (zinc finger CCCH-type containing 12A), TTP (Tristetraprolin), miR (microRNA).

## 4 Active resolution of inflammation

Cell death and removal of inflammatory leukocytes are the main drivers of resolution, offering a wealth of potential targets for developing pro-resolution therapeutics. Active resolution of inflammation involves: 1) death and clearance of inflammatory leukocytes; 2) functional macrophage switch; and 3) production of pro-resolution and tissue repair mediators. During resolution phase, regardless of whether the initial response was driven by PMNs, eosinophils, or lymphocytes responding to recall antigens, immune cells are cleared from inflamed tissues (Buckley et al., 2014). While some inflammatory leukocytes may return to systemic circulation, many PMNs, eosinophils and lymphocytes undergo local apoptosis or necrosis, followed by engulfing apoptotic cells by monocyte-derived macrophages, a process known as efferocytosis (Buckley et al., 2013). This process is governed by complex signaling pathways involving cell-to-cell receptor interactions and humoral mediators, notably bioactive lipids. After completing neutrophil apoptosis and efferocytosis, a shift towards a pro-resolution profile is triggered by effector cells including macrophages via releasing pro-resolving mediators (Serhan and Savill, 2005).

### 4.1 Death and clearance of inflammatory leukocytes

Three primary means by which inflammatory cells can be cleared from inflammatory sites: 1) retro-transendothelial migration back into systemic circulation, 2) lymphatic drainage, facilitating their role in adaptive immunity, and 3) cell death within inflamed tissues (Fullerton and Gilroy, 2016).

Many mechanisms, including autophagy, pyroptosis, necrosis, apoptosis and necroptosis mediate localized cell death (Fullerton and Gilroy, 2016). Granulocytes (e.g., neutrophils) are known to have a short lifespan, though pro-inflammatory cytokines (e.g., IL-6, IL-8 and GM-CSF) enhance survival of these cells. Likewise, eosinophil endurance is promoted by IL-3, IL-5 and GM-CSF (Filep, 2022). Pathways such as NF-κB enhance pro-survival molecules of granulocytes while reducing pro-apoptotic ones. Downregulating these mediators via dampening pro-inflammatory pathways is the initial step in activating pro-apoptotic pathways in inflammatory cells. The regulation of cell death pathways plays a crucial role in inflammation and its resolution, with several key mechanisms and signaling pathways influencing leukocyte survival. Inducing apoptotic pathways in inflammatory cells like PMNs, eosinophils and T helper (Th)1 lymphocytes can aid in resolving chronic inflammation. However, this strategy requires precise targeting to avoid disrupting pro-resolving and homeostatic cells (Alessandri et al., 2011; Leitch et al., 2012). Granulocyte apoptosis during resolution has been extensively studied, with key molecules and pathways regulating leukocyte survival or death, including CDK, NF-κB, PI3K–AKT, cAMP and MAPK. The following summarizes some of these important pathways and potential therapeutic targets.

#### 4.1.1 NF-κB pathway

NF-κB is a critical transcription factor involved in regulating expression of several pro-inflammatory mediators. Activation of NF-κB pathway influences cellular survival by regulating intracellular pro-survival proteins, including BCL-2 family members like BCL-XL and XIAP. Pharmacological inhibition of NF-κB and IKKα can enhance granulocyte apoptosis, promoting inflammatory resolution (Savill, 1997; Sousa et al., 2009; Lawrence and Fong, 2010; Remijsen et al., 2011; Vanden Berghe et al., 2014).

#### 4.1.2 cAMP pathway

Cyclic AMP (cAMP) plays a dual role in apoptosis, with its effects varying based on the context and cell type. cAMP was shown to inhibit granulocyte apoptosis and impair efferocytosis *in vitro* (Rossi et al., 1998; Lawrence and Fong, 2010). However, *in vivo* studies demonstrate that therapeutic increase of cAMP, via rolipram or forskolin, in LPS-induced pleurisy promotes PMN apoptosis and inhibits neutrophil accumulation (Sousa et al., 2010). Additionally, rolipram induces a cAMP-mediated switch of M1-like macrophages to M2 pro-resolving macrophages, highlighting cAMP’s therapeutic potential in regulating inflammation (Bystrom et al., 2008).

#### 4.1.3 PI3K–AKT pathway

The pro-apoptotic protein BAD, a downstream target of PI3K–AKT pathway, is phosphorylated by AKT to suppress apoptosis and promote cell survival (Song et al., 2005). In experimental autoimmune encephalomyelitis (EAE), a multiple sclerosis model, PI3Kγ-deficient mice showed reduced disease severity, lower CCL2 and CCL5 levels in brain tissue, and increased leukocyte apoptosis compared to wild-type controls (Rodrigues et al., 2010). Notably, neither PI3Kγ deficiency nor its pharmacological inhibition (e.g., with AS-605240) affected the acute phase of disease but instead reduced inflammation by promoting leukocyte apoptosis.

#### 4.1.4 MAPK pathway

Mitogen-activated protein kinase (MAPK) pathways have various roles in inflammation resolution ranging from enhancing neutrophil apoptosis to inducing polarization of anti-inflammatory macrophages. MAPK subtypes (e.g., ERK1/2, JNK, p38 MAPK) are involved in regulating cellular apoptosis (Junttila et al., 2008). ERK1/2 is typically linked to cell survival, but its inhibition can promote resolution of pleurisy by triggering PMNs apoptosis (Sawatzky et al., 2006; Chapman and Miner, 2011). Controversial roles of p38 MAPK were reported, with evidence for both pro-apoptotic (Langereis et al., 2010) and anti-apoptotic (Allaeys et al., 2014) effects. MAPK phosphatase 1 (MKP-1), an enzyme that regulates activity of MAPK, limits activation of p38 MAPK, aiding macrophage polarization and release of anti-inflammatory cytokines (Perdiguero et al., 2012).

#### 4.1.5 CDK inhibition

Cyclin-dependent kinase (CDK), which generally promotes cell survival by driving cell cycle progression, is increasingly recognized as emerging therapeutic tool prompting inflammation resolution (Rossi et al., 2006). By targeting CDK, therapies facilitate efferocytosis and enhance clearing of inflammatory cells. For example, inhibitors such as roscovitine induce apoptosis in granulocytes, enhancing resolution in GM-CSF- and LPS- stimulated human PMNs (Leitch et al., 2010; Perdiguero et al., 2012).

Therapeutic inducing of apoptosis is a promising strategy, however, effective efferocytosis is further required to prevent accumulation of apoptotic cells (Leitch et al., 2010). On the other hand, efferocytosis is an immunosuppressive process, with the potential to increase susceptibility to infections (Medeiros et al., 2009). Understanding interactions between apoptotic cells, phagocytosis and soluble mediators regulating these processes is essential for interpreting mechanisms underlying chronic inflammatory diseases and developing therapies. Efferocytosis is mediated by complex interplay of signals and receptors that assist in recognizing and engulfing apoptotic cells. Apoptotic PMNs release “find-me” signals such as ATP, which activate purinergic receptors on macrophages (Elliott et al., 2009). ICAM3 (CD50) binding to CD14 on macrophages and thrombospondin (TSP1) engaging CD36, further enhance apoptotic cell recognition (Savill et al., 1992; Moffatt et al., 1999). Lysophosphatidylcholine (LPC) and sphingosine-1-phosphate (S1P) interact with G-protein-coupled receptors G2A and S1P1-5, respectively, to guide phagocytes toward apoptotic PMNs (Lauber et al., 2003; Gude et al., 2008). In addition to “find-me” signal, “eat-me” signal (e.g., phosphatidylserine; PS) is crucial for specific recognition of apoptotic cells. Externalization of PS during apoptosis allows direct or opsonin-mediated binding to phagocytic receptors including αvβ3 (Poon et al., 2014; Elliott and Ravichandran, 2016).

Bridging molecules are essential for connecting macrophages and apoptotic cells. For example, αvβ3 or αvβ5 integrins on macrophages and PS on apoptotic cells are bound by the milk fat globule-EGF factor 8 protein (MFG-E8) (Hanayama et al., 2002). Likewise, developmental endothelial locus-1 (DEL-1) facilitates efferocytosis and inflammation resolution by connecting PS to αvβ3 integrins (Hajishengallis and Chavakis, 2019). Annexin A1 (ANXA1), galectin-3 and protein S are additional bridging molecules that promote PS-dependent clearance of apoptotic cells by interacting with TAM receptors (e.g., Tyro-3, Axl, Mer) (Anderson et al., 2003; Arur et al., 2003; Lemke and Burstyn-Cohen, 2010; Caberoy et al., 2012). By identifying PS, macrophage phagocytic receptors such TIM-1, TIM-4 and stabilin-2 aid in efferocytosis (Kobayashi et al., 2007; Park et al., 2008). Additional receptors, including CD14, scavenger receptor CD36 and integrin CD11b/CD18, are involved in regulating apoptotic cell clearance (Devitt et al., 1998; Mevorach et al., 1998). Defective clearance of apoptotic cells is associated with autoimmune disorders (Shao and Cohen, 2011) and chronic inflammatory diseases (Brown et al., 2003), making efferocytosis a critical process for resolving inflammation and maintaining immune tolerance (Brown et al., 2003).

### 4.2 Functional macrophage switch

**FIGURE 2:**
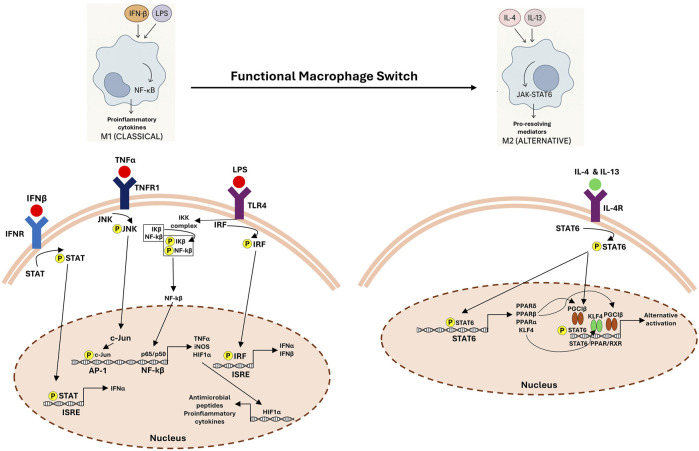
Functional macrophage switch: signaling pathways in M1 and M2 polarization. The left panel shows classical (M1) macrophage activation driven by lipopolysaccharide (LPS), tumor necrosis factor-alpha (TNF-α) and interferon-β (IFN-β), which activate Toll-like receptor 4 (TLR4), tumor necrosis factor receptor (TNFR) and interferon receptor (IFNR), respectively. This leads to downstream activation of STAT, JNK, IRF, and NF-κB signaling cascades, promoting the transcription of proinflammatory cytokines (e.g., TNFα), inducible nitric oxide synthase (iNOS), and antimicrobial peptides. The right panel depicts alternative (M2) activation mediated by interleukin-4 (IL-4) and interleukin-13 (IL-13) via IL-4 receptor (IL-4R), resulting in STAT6 phosphorylation and induction of anti-inflammatory and tissue repair-associated genes through PPAR and KLF4-dependent pathways. This switch supports the resolution of inflammation and tissue remodeling.

By triggering immunological reactions through releasing pro-inflammatory mediators, classically activated macrophages (M1) act as early respondents in inflammatory sites (Soliman and Barreda, 2022). However, these cells shift to alternatively activated anti-inflammatory (M2) phenotype during the resolution phase, which is necessary to eliminate apoptotic cells and facilitate resolution. Efferocytosis enables this transition, resulting in a decrease in pro-inflammatory cytokines and increased levels of anti-inflammatory mediators such as IL-10 and TGF-β (Soliman and Barreda, 2022). Several mediators and signaling pathways regulating the functional switch of macrophages from M1 (classical) to M2 (alternative) phenotype are summarized in Figure 2.

Prostaglandin E2 (PGE2) plays a complex role in inflammation, initially acting as a pro-inflammatory mediator by promoting vasodilation, edema and neutrophil recruitment during the onset phase (Ricciotti and FitzGerald, 2011). This is mediated through COX-2-derived PGE2, which is a key target of Nonsteroidal anti-inflammatory drugs (NSAIDs). However, during resolution, PGE2 contributes to macrophage reprogramming by suppressing TNF-α and IL-1β production while enhancing IL-10 release, thereby facilitating the transition to a pro-resolving environment (Ricciotti and FitzGerald, 2011). PGE2 further reduces synthesis and secretion of TNF-α and IL-1β from macrophages (Kunkel et al., 1986; Katakami et al., 1988). This oppressive effect allows PGE2 to act as self-regulator of pro-inflammatory cytokines (Kunkel et al., 1986). Recently, PGE2 has been shown to enhance IL-10 release in LPS-stimulated macrophages (MacKenzie et al., 2013). PGE2 induces production of IL-10 in alternatively active macrophages through EP receptor signaling, suggesting a continuous role in macrophage reprogramming (Németh et al., 2009). Others reported that PGE2 displays a bipolar effect on IL-6 transcription, promoting its expression initially before limiting it (MacKenzie et al., 2013). High levels of PGE2 reduced mucosal inflammation and colitis symptoms in DSS-induced colitis murine model (Zhang Y. et al., 2015). In lungs, PGE2 was released by fibroblasts and alveolar macrophages, limiting inflammatory reactions and facilitating tissue repair (Vancheri et al., 2004). Conversely, low levels of PGE2 have been linked to lung fibrosis in human and animal models, highlighting its role in effective inflammation resolution (Wilborn et al., 1995; Hodges et al., 2004).

Macrophages undergo metabolic adaptations as they process the metabolites of apoptotic cells, influencing their pro-resolving functions (Schilperoort et al., 2023b). Apoptotic cell-derived arginine facilitates multiple cycles of efferocytosis, termed continual efferocytosis, in murine macrophages generated *in vitro* or isolated during resolution (Yurdagul et al., 2020). In contrast, human monocyte-derived macrophages primarily utilize apoptotic cell-derived ornithine to sustain continual efferocytosis (Yurdagul et al., 2020). These findings highlight cellular metabolic dependencies, underscoring the necessity of mechanistic investigations of immunometabolism in human macrophages. Macrophages engaging in efferocytosis activate distinct metabolic pathways that enhance production of pro-resolving mediators (Zhang et al., 2019). For example, suppression of *Cpt1*, which encodes a key enzyme in mitochondrial long-chain fatty acid oxidation, in murine macrophages co-cultured with apoptotic cells, led to decreased IL-10 levels. Interestingly, when glycolysis was inhibited, IL-10 production remained unaffected (Zhang et al., 2019). These findings indicate that fatty acid oxidation is a crucial metabolic pathway for efferocytosis-driven IL-10 production in macrophages. Other reports indicated that glycolysis is non-replaceable for definite efferocytosis persistence in murine as well as human macrophages (Schilperoort et al., 2023a).

Additionally, GLS1-modified by glutaminase 1 (GLS1)-mediated glutaminolysis was required for established efferocytosis. Macrophages that were removed from liver and spleens of *Gls1*-deficient mice were identified to be defective in efferocytosis when they were subsequently challenged with labeled apoptotic cells (Merlin et al., 2021). In atherosclerosis murine model, efferocytosis was impaired in *Gls1*-deficient mice, facilitating the deterioration of disease (Merlin et al., 2021). Interestingly, macrophages no matter if differentiated *in vitro* or isolated from tissues (e.g., spleen and thymus), maintained clearing of apoptotic cells under hypoxic conditions by developing the required metabolic profile (Wang et al., 2023). Table 2 summarizes various drugs and compounds that promote M2 macrophage polarization by targeting specific signaling pathways. These interventions have been studied in different experimental models, demonstrating their potential to modulate inflammation and enhance tissue repair. Future studies should investigate how efferocytosis of cells undergoing pro-inflammatory cell death (i.e., pyroptosis, necroptosis, ferroptosis) might impact macrophage-mediated resolution of inflammation.

**TABLE 2:** Recent advances in targeting macrophage phenotype transitions during inflammation resolution.

Drug/compound	Target pathway	Effect	Model	References
Resolvin D1	GPR32/ALX/FPR2 receptors	Promotes M2 polarization, enhances efferocytosis	Human macrophages	Arnardottir et al. (2021)
Maresin 1	LGR6 receptor	Induces M2 phenotype, reduces inflammation	Mouse peritonitis model	Zhao et al. (2022)
IL-4	IL-4 receptor α	Enhances M2 polarization, accelerates resolution	Murine muscle injury	Weng et al. (2018)
IL-10	STAT3 pathway	Promotes M2 phenotype, suppresses inflammation	Human monocytes	Xia et al. (2023)
TGF-β1	SMAD2/3 pathway	Induces M2 polarization, enhances tissue repair	Murine lung injury	Zhang et al. (2016)
PPARγ Agonist (Rosiglitazone)	PPARγ pathway	Promotes M2 phenotype, reduces inflammation	Mouse colitis	Xue and Wu (2025)
IL-13	IL-4 receptor α	Enhances M2 polarization, promotes tissue repair	Murine skin wound healing	Hassanshahi et al. (2022)
Glucocorticoids (dexamethasone)	Glucocorticoid receptor	Induces M2 phenotype, suppresses inflammation	Human macrophages	Xie et al. (2019)
IL-33	ST2 receptor	Promotes M2 polarization, enhances efferocytosis	Mouse atherosclerosis model	Sheng et al. (2018)
Vitamin D3	Vitamin D receptor/MAPK	Blocks M1 phenotype, reduces inflammation	Human monocytes	Zhang et al. (2012)
LXA_4_	12/15-lipoxygenase	Induces M2 phenotype, suppresses inflammation	Murine arthritis	Jackson et al. (2023)
Mesenchymal stem cells	Paracrine signaling	Enhances M2 phenotype, suppresses inflammation	Mouse lung injury	Ionescu et al. (2012)
PGE2	EP2/EP4 receptor	Induces M2 phenotype, suppresses inflammation	Human macrophages	Wu et al. (2018)
IL-6	IL-6 receptor	Promotes M2-like phenotype, enhances resolution	Murine colitis	Zhang et al. (2023)
Omega-3 fatty acids	NF-κB inhibition	Enhances M2 phenotype, reduces inflammation	Human monocytes	Videla et al. (2023)

GPR32 (G protein-coupled receptor 32), ALX/FPR2 (lipoxin A4/formyl peptide receptor 2), LGR6 (leucine-rich repeat-containing G-protein-coupled receptor 6), IL (interleukin), STAT3 (signal transducer and activator of transcription 3), TGF-β1 (transforming growth factor beta 1), SMAD2/3 (SMAD, family members 2 and 3), PPARγ (peroxisome proliferator-activated receptor gamma), ST2 (suppression of tumorigenicity 2 receptor), MAPK (mitogen-activated protein kinase), LXA_4_ (lipoxin A4), EP2/EP4 (prostaglandin E2 receptors 2 and 4), NF-κB (nuclear factor kappa B), PGE2: Prostaglandin E2.

### 4.3 Production of pro-resolution and tissue repair mediators

**FIGURE 3:**
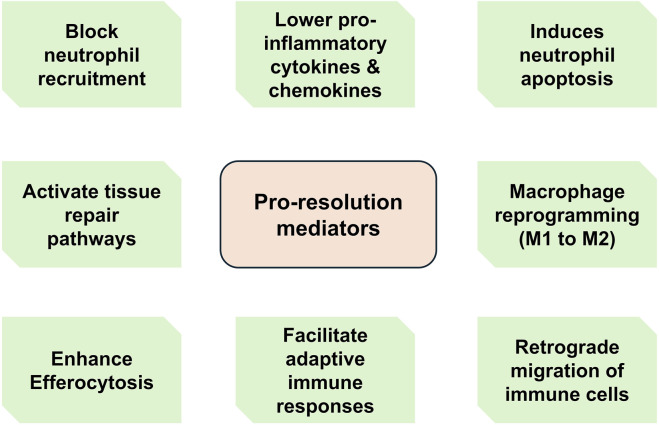
Main functions of pro-resolution mediators. During active resolution, a variety of effector cells including macrophages secrete pro-resolving molecules such as specialized pro-resolving mediators (SPMs). These molecules mediate various functions ranging from suppressing granulocyte recruitment to regulating tissue repair pathways and facilitating adaptive immune responses.

**FIGURE 4:**
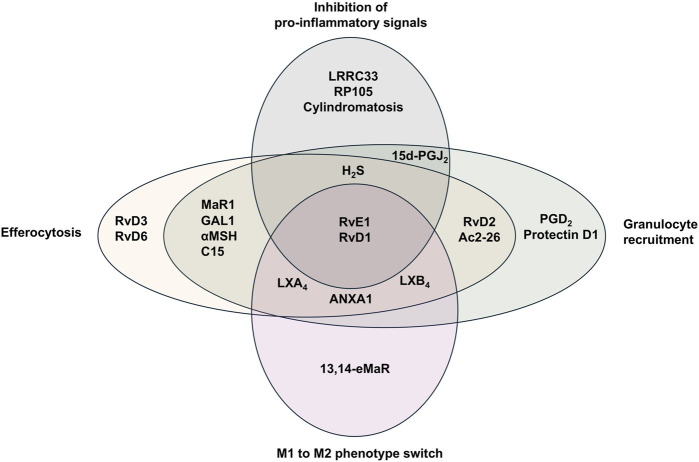
Specialized pro-resolving mediators and their reported functions during resolution of inflammation. Specialized pro-resolving mediators (SPMs) mediate several effector functions during resolution of inflammation. LX (lipoxin), Rv (resolvin), ANXA1 (annexin A1), PGD (prostaglandin D), 15d-PGJ2 (15-deoxy-Δ12,14-prostaglandin J2), MaR1 (maresin 1), αMSH (alpha-melanocyte-stimulating hormone), C15 (chemerin-derived peptide), H_2_S (hydrogen sulfide).

Macrophages engulfing apoptotic cells shift toward a pro-resolution phenotype (Junttila et al., 2008), that is characterized by increased expression of inhibitory molecules (e.g., PDL1 and ICOS ligand), secretion of anti-inflammatory cytokines, and reduced release of pro-inflammatory cytokines (e.g., TNF, IL-1β, IL-18) (Poon et al., 2014). They also produce pro-resolving lipid mediators, including SPMs (Schwab et al., 2007; Serhan et al., 2012), which contribute to many steps of resolving inflammation and subsequent restoration of tissue homeostasis (Figure 3). Table 3 details to a large extent pro-resolution mediators and their mechanisms of action in overcoming inflammation. SPMs, including lipoxins, resolvins, maresins and protectins, play a crucial role in actively resolving inflammation (Figure 4). These mediators not only counteract excessive immune activation but also promote tissue repair and immune balance (Serhan and Levy, 2018). The exact mechanisms by which SPMs exert their anti-inflammatory and pro-resolving effects is not fully understood. SPMs promote inflammation resolution as well as switch the balance from effector to regulatory T cell (T_reg_). Other SPMs such as protectins, maresins, and D-series resolvins have been shown to function as biased positive allosteric modulators (PAM) of the prostaglandin E2 (PGE2) receptor (EP4), promoting anti-inflammatory signaling of EP4 (Alnouri et al., 2024).

**TABLE 3:** Pro-resolution mediators and their mechanistic contributions to inflammation resolution.

Pro-resolution functions	Mediator	Receptor	Target/mechanism	References
Inhibition of granulocyte recruitment to inflammation site	LXA_4,_ LXB_4_	FPR2, GPR32	PMNs, NK cells	Chiang et al. (2006), Barnig et al. (2013)
	RvD1		PMNs, Macrophages	Xiang et al. (2021)
	ANXA1		PMNs, Eosinophils	Cirino et al. (1993), Wang et al. (2011), Perretti and Dalli (2023)
	PGD2	DP1, DP2	Eosinophils	Ward et al. (2002)
	15d-PGJ2	PPARγ	PMNs	Napimoga et al. (2008)
	MaR1	ND	PMNs, Eosinophils	Serhan et al. (2012), Ou et al. (2021)
	Protectin D1	ND	PMNs, T cells, Macrophages	Ariel et al. (2005), Nguyen-Chi et al. (2020)
	RvE1	BLT1, CMKLR1	Dendritic cells	Sawada et al. (2015)
	RvD2	GPR18	PMNs	Gao et al. (2023a)
	Galectin 1	CD3, CD7, CD43, CD45	PMNs, Eosinophils	Auvynet et al. (2013), Rao et al. (2017), Law et al. (2020)
	αMSH	MC3R	PMNs	Brzoska et al. (2010)
	C15	CMKLR1	PMNs	Cash et al. (2013)
Pro-inflammatory cytokine scavenging	LXA_4_	FPR2	Reduces CD11b/CD18 expression, NF-κB activation	Bäck et al. (2014)
	D6	N/A	D6 acts as a decoy receptor	Graham and Locati (2013)
Inhibition of pro-inflammatory signaling pathways	15d-PGJ2	PPARγ	NO-dependent reduction in leukocyte rolling and adhesion	Napimoga et al. (2008)
	H_2_S	ND	Inhibits NF-kB, activates Nrf-2 molecular pathways	Benedetti et al. (2017)
	RvE1	BLT1, CMKLR1	Inhibits TNF-α-induced nuclear translocation of NF-κB	Ishida et al. (2010)
	RvD1	FPR2, GPR32	Stimulates phosphorylation of AKT, prevents cleavage of caspase-3	Xie et al. (2016)
Efferocytosis	αMSH	MC3R	Apoptotic PMNs	Wallace and Wang (2015)
	LXA_4,_LXB_4_	FPR2, GPR32	Apoptotic PMNs	Mitchell et al. (2002), Maderna et al. (2010), Bäck et al. (2014)
	RvD1		Apoptotic PMNs via p50/p50-homodimer-mediated repression of TNF expression	Lee et al. (2013)
	ANXA1		Ac2-26 stimulates phagocytosis of apoptotic PMNs	Maderna et al. (2005)
	RvD2	GPR18	RvD2/GPR18 axis regulates polarization and efferocytosis through activation of PI3K/Akt	Zhao et al. (2023)
	MaR1	ND	Redirects macrophage activation toward M2 phenotype, increases phagocytic engulfment of PMNs via silencing pro-inflammatory intracellular signaling (STAT3, ERK1/2)	Francos-Quijorna et al. (2017)
	Galectin 3	CD3, CD7, CD43, CD45	Enhances monocyte-derived macrophage efferocytosis of apoptotic PMNs	Erriah et al. (2019)
	RvE1	BLT1, CMKLR1	Facilitates PMNs apoptosis and their removal	El Kebir et al. (2012)
M1 to M2 polarization	LXA_4,_ LXB_4_	FPR	Polarization of THP-1-derived macrophages	Amici et al. (2017), Aubeux et al. (2023)
	RvD1		Promotes anti-inflammatory M2 phenotype, enhances phagocytic function of recruited macrophages	Xiang et al. (2021)
	ANXA1		AnxA1/FPR2/AMPK axis	Vago et al. (2012), McArthur et al. (2020)
	RvE1	BLT1, CMKLR1	RvE1 inhibits LPS-mediated M1 macrophage polarization, promotes polarization toward M2 phenotype	Zhang et al. (2020)
	H_2_S	ND	Promotes mitochondrial biogenesis and FAO, inhibits iNOS, NF-κB, ERK, p38 MAPK signaling pathways	Zhang et al. (2014), Miao et al. (2016), Sun et al. (2021)

LXA (lipoxin A), FPR2 (formyl peptide receptor 2), GPR32 (G protein-coupled receptor 32), PMNs (polymorphonuclear neutrophils), NK, cells (natural killer cells), RvD1 (resolvin D1), ANXA1 (annexin A1), PGD2 (prostaglandin D2), DP1/DP2 (prostaglandin D2 receptors 1,2), 15d-PGJ2 (15-deoxy-Δ12, 14-prostaglandin J2), PPARγ (peroxisome proliferator-activated receptor gamma), MaR1 (maresin 1), ND (not determined), BLT1 (leukotriene B4 receptor 1), CMKLR1 (chemerin receptor 1), RvE1 (resolvin E1), RvD2 (resolvin D2), GPR18 (G protein-coupled receptor 18), Galectin 1 (lectin family protein involved in immune regulation), αMSH (alpha-melanocyte-stimulating hormone), MC3R (melanocortin receptor 3), C15 (chemerin-derived peptide), D6 (atypical chemokine receptor acting as a decoy receptor), NO (nitric oxide), H_2_S (hydrogen sulfide), Nrf-2 (nuclear factor erythroid 2-related factor 2), TNF-α (tumor necrosis factor-alpha), AKT (protein kinase B), PI3K (phosphoinositide 3-kinase), Ac2-26 (annexin A1-derived peptide), STAT3 (signal transducer and activator of transcription 3), p38 MAPK (p38 mitogen-activated protein kinase), ERK (extracellular signal-regulated kinase), Galectin 3 (a lectin involved in immune regulation), FAO (fatty acid oxidation), FPR (formyl peptide receptor), THP-1 (human monocytic cell line), AMPK (AMP-activated protein kinase), LPS (lipopolysaccharide).

Resolvin E4 (RvE4), a newly identified member of resolvin family, has been reported to enhance efferocytosis (Serhan and Levy, 2018). Meanwhile, RvE1 reduces differentiation of Th17 and thus IL-17 production (Oner et al., 2021). RvD1 suppresses inflammation via post-transcriptional regulation, linking miRNA modulation to macrophage reprogramming (Amin Mohedin et al., 2024). Explicitly, RvD1 prevented age-related inflammation by reducing macrophage senescence and fibrosis in spleens of aged mice, indicating its anti-aging and anti-fibrotic potential (Groenen et al., 2024). Precise delivery of RvD1 using nanotechnology enhanced its anti-inflammatory effects within vascular lesions (He et al., 2024). RvD2 activation of GPR18 on macrophages reduces plaque burden and inflammation in atherosclerosis murine models, showing targeted SPM signaling benefits (Lipscomb et al., 2024). Likewise RvT4 promotes macrophage cholesterol efflux, reducing atherosclerosis and highlighting a novel role for SPMs in lipid homeostasis in cardiovascular diseases (Walker et al., 2024). Mechanistically, resolvin enhances mitochondrial metabolism and reduces inflammatory gene expression, supporting long-term macrophage function under inflammatory stress (Padovani et al., 2024). Additionally, resolvins demonstrated diagnostic and prognostic potentials in experimental models and clinical trials (Perez-Hernandez et al., 2021). A clinical study demonstrated that low RvD1 and LXA_4_ levels correlated with inflammation severity in gallstone-induced pancreatitis patients (Mısırlıoglu et al., 2025).

SPMs exhibit various pro-resolution effects in organ-specific inflammation and injury. RvD4 mitigates LPS-induced lung injury via reducing neutrophil infiltration and cytokine levels (Inomata et al., 2024). RvE1 has been also shown to protect against one-lung ventilation-induced injury via diminishing cytokine storms and epithelial damage (Ji et al., 2024). In pressure-overload-induced heart failure model, RvD2/GPR18 axis ameliorated pro-inflammatory macrophage polarization (Zheng et al., 2024). RvD1 with exercise therapy was reported to mediate neuroprotective effects in intracranial hemorrhage mice models via BDNF/TrkB/PI3K/AKT signaling by enhancing neural recovery post-stroke (Xiaoyu et al., 2024). RvD2 induces dentin regeneration and pulp stem cell activation post-pulpotomy, showcasing dental regenerative potential of SPMs through stimulation of reparative processes in dental tissues (Yoneda et al., 2024).

SPMs have been found to change tumor-associated macrophages (TAMs) into an anti-tumor phenotype in cancer (Lavy et al., 2021). SPMs also include Protectin Conjugates in Tissue Regeneration 1 (PCTR1) and Protectin D1 (PD1). PCTR1 and PD1 were detected during the resolution phase of respiratory synthetical virus (RSV) infection where they demonstrated modulatory effects on secondary immune responses in lungs (Walker et al., 2021). Similarly, RvD1 inhibited IL-6/STAT3-driven epithelial mesenchymal transmission in colorectal cancer, suppressing cancer progression by interfering with inflammatory cytokine signaling (Du et al., 2024).

Maresin 1 (MaR1), a lipid mediator derived from docosahexaenoic acid, plays a crucial role in the resolution of inflammation by promoting macrophage-mediated efferocytosis and reducing pro-inflammatory cytokine production (Francos-Quijorna et al., 2017). Dysregulated maresin 1 production was associated with chronic rhinosinusitis. Maresin 1 is also involved in the regulation of adhesion molecule expression in phagocytes, resulting in promoting inflammation resolution (Beegun et al., 2021). Prostanoids, which include prostaglandins and thromboxanes, fuel the immune system to start normally but then induce inflammation resolution. Specifically, PGE2 and PGD2 affect immune cell activity by regulating neutrophil influx, enhancing macrophage-mediated clearance of apoptotic cells, and shifting from a pro-inflammatory to a reparative environment. These bioactive lipids are prominent factors leading the shift from inflammation to resolution, and as a result, they are promising therapeutic candidates for inflammatory diseases (Schmid and Brüne, 2021). Likewise, RvE1 enhances macrophage efferocytosis and restores hematopoiesis in aplastic anemia, suggesting a therapeutic role of RvE1 in bone marrow failure syndromes (Grazda et al., 2024). RvE1 and MaR1 enhance osteogenesis of bone marrow stem cells under inflammatory stress by resolving inflammation and promoting regenerative capacity through enhancing osteoblast differentiation (Alzahrani et al., 2024).

Recent studies indicated phytocannabinoids, like cannabidiol (CBD) and tetrahydrocannabinol (THC), as key modulators of inflammation resolution. CBD and THC activate the endocannabinoids system (ECS) through CB1 and CB2 receptors, triggering the endocannabinoid pathway. This system influences immune cell function, suppresses the production of pro-inflammatory mediators and promotes M2 macrophage efferocytosis (Nagarkatti et al., 2009; Burstein, 2015). Besides, phytocannabinoid treatment increases apoptosis and promotes tissue repair by suppressing oxidative stress and downregulating fibrotic activity. Immunomodulatory properties of these substances have made them favorable for utilizing them in chronic inflammatory diseases and infections, even in extreme cases such as COVID-19 (Paland et al., 2021).

## 5 Post-resolution

The post-resolution phase bridges innate and adaptive immunity, where molecular pathways regulating resolution events (e.g., macrophage reprogramming) directly influence long-term immune memory and tissue repair. For instance, resolvins and maresins have been found to not only assist in clearing apoptotic neutrophils but also modulating dendritic cell (DCs) trafficking to lymph nodes and priming T-cell responses (Chiurchiù et al., 2016). Subsequently, macrophages transitioning to an M2 phenotype secrete TGF-β and IL-10, which dampen inflammation while promoting T_reg_ expansion, a critical step in preventing autoimmune reactivation (Chen et al., 2023). This crosstalk underscores how resolution pathways are not merely endpoints but active participants in immune homeostasis, with failures leading to chronic inflammation or fibrosis.

The traditional view that resolution marks the end of inflammatory responses is increasingly challenged by recent reports confirming a post-resolution phase (Newson et al., 2014). It was reported that after resolution of innate immune responses to fungal mimics (zymosan) or bacterial infection (*S. pneumoniae*), a second wave of leukocytes infiltrated tissues, consisting of monocyte-derived macrophages, myeloid-derived suppressor cells, DCs and macrophages (Ersland et al., 2010; Newson et al., 2014). Furthermore, tissue-resident embryonically derived macrophages, previously lost during acute inflammation, re-emerged (Blériot et al., 2020). These populations were further associated with increased memory T and B lymphocytes in tissues and absence of PMNs (Nakano et al., 2009; Ersland et al., 2010). Collectively, the data suggest that immune cells originating from resolving tissues or peripheral blood enhance adaptive immune responses during resolution by persisting in tissues for months post-inflammation (Yona et al., 2013; Panigrahy et al., 2021). Thus, these post-resolution processes shape a connection between innate and adaptive immunity, where induction of acute inflammation and its resolution are crucial for regulating humoral and cell-mediated immune responses.

Post-resolution is crucial for establishing long-term immune memory and ensuring effective tissue remodeling. SPMs, such as resolvins, protectins and maresins are not merely passive byproducts but actively orchestrate the cessation of inflammation, promote clearance of inflammatory cells and facilitate tissue repair and regeneration (Spite et al., 2014). The phase is thus crucial for restoring tissue homeostasis and preventing chronic inflammation, which can lead to fibrosis and impaired function. SPMs modulate adaptive immunity by influencing DCs function and T cell responses (Serhan and Levy, 2018). This modulation ensures that the immune system can remember and respond more effectively to future insults, thereby linking the resolution of inflammation directly to quality and durability of adaptive immune memory.

## 6 Epigenetic regulatory mechanisms during inflammation resolution

Emerging evidence highlights epigenetic mechanisms as pivotal regulators of inflammation resolution, primarily through the modulation of gene expression in innate immune cells (Zhang and Cao, 2021). Histone modifications, such as methylation and acetylation, dynamically regulate inflammatory gene transcription in macrophages and DCs. These modifications influence the accessibility of transcriptional machinery to gene of pro- and anti-inflammatory cytokines, thereby controlling the intensity and duration of immune responses (Zhang and Cao, 2021). For instance, histone demethylase Tet2 plays a crucial role in repressing IL-6 transcription during resolution phase, preventing prolonged inflammation and promoting tissue homeostasis (Zhang Q. et al., 2015).

Macrophage polarization is another critical aspect influenced by epigenetic regulation. Epigenetic modifications govern the transition between pro-inflammatory M1 and anti-inflammatory M2 macrophage phenotypes (Kapellos and Iqbal, 2016). DNA methylation (e.g., DNMT3A-mediated silencing of pro-inflammatory genes) and histone modifications (e.g., HDAC3-dependent deacetylation) dynamically modulate macrophage polarization (Jin et al., 2021). Jin et al. explored the potential of targeting epigenetic modifiers to reprogram macrophages in non-resolving inflammatory conditions, such as in atherosclerosis (Jin et al., 2021). By modulating epigenetic enzymes, it is possible to shift macrophage polarization towards a reparative phenotype, offering therapeutic avenues for chronic inflammatory diseases.

## 7 Translational research and therapeutic implications

Understanding the therapeutic potential of these pathways requires a detailed examination of how they can be targeted to accelerate inflammation resolution in clinical settings. Table 4 summarizes key molecular pathways involved in inflammation resolution, highlighting their downstream targets, effects on resolution, and the progress made in testing these pathways as therapeutic targets. Targeting resolution pathways in chronic inflammatory conditions may yield valuable insights for resolution pharmacology. As preclinical research continues to elucidate the functions of SPMs in inflammation, their potential as therapeutic agents is drawing increasing interest. For example, RvD2 in combination with omega-3 polyunsaturated fatty acid mitigated inflammatory bowel disease (IBD) symptoms in murine models through modulation of intestinal inflammation and epithelial repair (Chaim et al., 2024). Recently imidazole-derived RvD1 analogues with antioxidant activity demonstrated promising potentials for the treatment of oxidative stress-related diseases, including osteoarthritis (Kariminezhad et al., 2024). Another study showed that RvD1 promotes chondrocyte proliferation in osteoarthritis via NLRP3/caspase-1 suppression, highlighting mechanistic insights into how RvD1 can reduce inflammasome activation and restore cartilage cell health (Wang et al., 2024). RvD1 suppresses inflammation in synoviocytes via p38, NF-κB, and AKT pathways, supporting the use of RvD1 in treating inflammatory joint diseases like rheumatoid arthritis (Yanoshita et al., 2025). Ongoing clinical trials are investigating the efficacy of SPMs-based synthetic analogs, designed to resist metabolic degradation and extend their pharmacological effects. These analogs are being evaluated for their therapeutic potential in inflammatory diseases, including dry eye, asthma, periodontitis, inflammatory bowel disease and cardiovascular disorders. Table 5 summarizes key translational research efforts exploring SPMs interventions in clinical settings, highlighting their study outcomes and therapeutic potential.

**TABLE 4:** Molecular pathways in inflammation resolution and their therapeutic implications.

Pathway	Target	Receptor	Mechanism	Implications	References
Vagus nerve stimulation (VNS)	Gut-brain axis, immune Cells	N/A	VNS modulates Gut-Brain Axis in patients with IBD, reducing inflammation	Reduces pain and diarrhea, balances immune activity	Hesampour et al. (2024)
IL-10 signaling pathway	Macrophages, M2-like phenotype	IL-10R	Enhances macrophage reprogramming to pro-resolving M2 phenotype	*In-vivo* infusion of IL-10 post-myocardial infarction suppresses inflammation and facilitates wound healing	Jung et al. (2017)
Galectin-1	T-cells, macrophages	N/A	Induces T-cell apoptosis, enhances macrophage efferocytosis	Gal-1 administration in murine models reduced inflammation and promoted immune tolerance	Iqbal et al. (2011), Yaseen et al. (2020)
Annexin A1	Macrophage, neutrophils	FPR1, FPR2	Promotes efferocytosis, downregulates PMNs infiltration	Targeting AnxA1/FPR2/ALX pathway protects against thromboinflammatory diseases	Ansari et al. (2021)
NLRP3 deactivation	IL-1β, IL-18	NLRP3	Reduces pro-inflammatory cytokine production, restores tissue homeostasis	Inhibiting NLRP3 reduces inflammation in animal models of chronic diseases	Coll et al. (2015), Mangan et al. (2018)
CX3CL1-CX3CR1 axis	Monocytes, macrophages	CX3CR1	Enhances macrophage recruitment, promotes efferocytosis	Promoting CX3CL1-CX3CR1 axis has been shown to modulate immune cell recruitment and improve resolution	Truman et al. (2008), Gao et al. (2023b), Szukiewicz (2024)
Nitric oxide (NO)	Neutrophils, macrophage, endothelial Cells	N/A	Reduces PMNs adhesion, enhances macrophage clearance, supports vascular repair	NO regulates clearance of apoptotic neutrophils and production of pro-inflammatory cytokines	Chello et al. (1998), Kobayashi (2010)

IBD (inflammatory bowel disease), IL (interleukin), IL-10R (interleukin 10 receptor), M2 (alternatively activated macrophages), Gal-1 (galectin-1), FPR (formyl peptide receptor), FPR1/FPR2 (formyl peptide receptors 1 and 2), PMNs (polymorphonuclear neutrophils), AnxA1 (annexin A1), ALX (lipoxin A4 receptor), NLRP3 (NOD-, LRR-, and pyrin domain-containing protein 3), NO (nitric oxide), N/A (not applicable).

**TABLE 5:** Translational research on specialized pro-resolving mediators (SPMs) and related therapeutic interventions.

Research area	Key findings	Clinical applications	Challenges & limitations	References
Omega-3 dietary interventions	Randomized controlled trials indicate that fatty acids lower inflammatory markers (IL-1β, IL-6, TNF-α, CRP)	Reduces myocardial infarction risk, modulates immune response in autoimmune diseases, improves periodontal healing	Variability in clinical responses, dosage standardization, bioavailability issues	Vedin et al. (2008), El-Sharkawy et al. (2010), Miles and Calder (2012), Hu et al. (2019)
LXA_4_ analogs	Stable LXA_4_ analogs (e.g., 15-(R/S)-methyl-LXA_4_) exhibit comparable efficacy to corticosteroids in eczema. BLXA_4_ promotes systemic pro-resolving pathways when applied topically	Targeting inflammatory diseases, including dry eye, asthma, periodontitis, inflammatory bowel diseases, cardiovascular diseases	Stability and delivery remain key challenges in clinical translation. Small sample sizes in early-phase trials; larger clinical trials are needed to confirm long-term efficacy and safety	Wu et al. (2013), Van Dyke et al. (2015), Hasturk et al. (2021)
RvE1 analog	RX-10045 (RvE1 analog) improves tear film stability in dry eye models, while BDA-RvD1 reduces neutrophilic inflammation in ischemia–reperfusion lung injury models	RX-10045 tested in phase 2 trials for dry eye and allergic uveitis; BDA-RvD1 investigated for acute lung inflammation	RX-10045 showed inconsistent efficacy; potential issues with drug permeability across the corneal epithelium	Cholkar et al. (2015), Orr et al. (2015), Resolve SARL (2019)
Non-lipid GPCR agonists	FPR2/ALX receptor agonists reduce immune cell degranulation	ACT-389949 studied in phase 2 trials; demonstrated transient cytokine suppression	Temporary leukocyte reduction observed; receptor desensitization with prolonged dosing	Wang et al. (2004), Stalder et al. (2017)

(Specialized Pro-Resolving Mediators), LXA (Lipoxin A), RvE1 (Resolvin E1), TNF-α (Tumor Necrosis Factor Alpha), CRP (C-Reactive Protein), FPR2/ALX (Formyl Peptide Receptor 2/Annexin A1 Receptor), GPCR (G Protein-Coupled Receptor), IL (Interleukin).

## 8 Conclusion

The resolution of inflammation is an active, highly regulated process that restores tissue homeostasis and prevents the transition to chronic inflammation. Over the past decades, significant progress has been made in elucidating the molecular and cellular mechanisms underlying resolution, including the roles of SPMs, macrophage reprogramming and efferocytosis. These insights have shifted the paradigm from traditional anti-inflammatory treatments to pro-resolution strategies, which offer a more targeted and physiological approach to managing chronic inflammatory diseases.

Despite these advances, challenges remain in translating fundamental discoveries into clinical therapies. The complexity of resolution pathways, their tissue-specific dynamics, and the interplay between innate and adaptive immunity necessitate further investigation. Emerging therapeutic approaches, including lipid mediator analogs, nanomedicine, and immunometabolic interventions, hold promises for enhancing resolution in disease contexts. Future research should focus on refining biomarker identification, optimizing drug delivery systems, and integrating computational models to advance resolution-targeted treatments. By leveraging these strategies, resolution pharmacology has the potential to revolutionize how inflammatory diseases are treated, shifting the focus from merely suppressing inflammation to actively restoring immune balance and tissue integrity.
